# Optical Gas Sensing of Ammonia and Amines Based on Protonated Porphyrin/TiO_2_ Composite Thin Films

**DOI:** 10.3390/s17010024

**Published:** 2016-12-23

**Authors:** Pedro Castillero, Javier Roales, Tânia Lopes-Costa, Juan R. Sánchez-Valencia, Angel Barranco, Agustín R. González-Elipe, José M. Pedrosa

**Affiliations:** 1 Instituto de Ciencia de Materiales de Sevilla, CSIC–Universidad de Sevilla, Américo Vespucio 49, Sevilla 41092, Spain; pcastillerod@hotmail.com (P.C.); jrsanchez@icmse.csic.es (J.R.S.-V.); angel.barranco@csic.es (A.B.); arge@icmse.csic.es (A.R.G.-E.); 2 Departamento de Sistemas Físicos, Químicos y Naturales, Universidad Pablo de Olavide, Ctra. Utrera Km. 1, Sevilla 41013, Spain; jroabat@upo.es (J.R.); tlopcos@upo.es (T.L.-C.)

**Keywords:** MMPyP, TMPyP, TiO_2_, GLAD-PVD, optically active composites, ammonia and amine gas sensing, porous thin films

## Abstract

Open porous and transparent microcolumnar structures of TiO_2_ prepared by physical vapour deposition in glancing angle configuration (GLAD-PVD) have been used as host matrices for two different fluorescent cationic porphyrins, 5-(*N*-methyl 4-pyridyl)-10,15,20-triphenyl porphine chloride (MMPyP) and meso-tetra (*N*-methyl 4-pyridyl) porphine tetrachloride (TMPyP). The porphyrins have been anchored by electrostatic interactions to the microcolumns by self-assembly through the dip-coating method. These porphyrin/TiO_2_ composites have been used as gas sensors for ammonia and amines through previous protonation of the porphyrin with HCl followed by subsequent exposure to the basic analyte. UV–vis absorption, emission, and time-resolved spectroscopies have been used to confirm the protonation–deprotonation of the two porphyrins and to follow their spectral changes in the presence of the analytes. The monocationic porphyrin has been found to be more sensible (up to 10 times) than its tetracationic counterpart. This result has been attributed to the different anchoring arrangements of the two porphyrins to the TiO_2_ surface and their different states of aggregation within the film. Finally, there was an observed decrease of the emission fluorescence intensity in consecutive cycles of exposure and recovery due to the formation of ammonium chloride inside the film.

## 1. Introduction

The detection and measurement of gas concentrations is important for understanding and monitoring a variety of phenomena, from industrial processes to environmental change. During the last decades, there has been a growing demand for the development of sensitive, portable, and cost-effective sensors for gases such as ammonia and amines. Ammonia plays an important role in several environmental issues, such as eutrophication or acidification processes, human health, and atmospheric particle formation, contributing to climate change [1]. Its detection is also of interest for medical applications [2,3]. Some amines appear as reaction intermediates in the production of chemicals, and their presence needs to be controlled because of their potential toxicity [4]. Other amines can be used as food freshness indicators, as they are present in rotting food as a result of bacterial decomposition [5,6,7,8].

The most common approaches for the detection of volatile organic compounds have been based on metal-oxide gas sensors [9,10,11], catalytic detectors [12,13], conducting polymer analysers [14,15], and optical detection techniques [13,16]. Metal-oxide gas sensors, mostly based on SnO_2_ or WO_3_ [9,17,18,19], are the most widely used for this purpose, showing limits of detection as low as 1 ppm. However, these sensors have the drawbacks of operating at an elevated temperature of more than 400 °C and being highly humidity-dependent [20]. An interesting alternative is the use of organic dyes, such as porphyrins, as active elements, which can work at room temperature [21,22] and can be optically monitored [23,24]. These sensors based on optical changes offer fast responses in the range of seconds and the possibility of real-time monitorisation [25,26], which is vital in industrial processes [27]. Besides, they offer a minimal drift and high gas specificity, filling an important gap between lower cost sensors with inferior performance and high-end laboratory equipment.

Porphyrins have been widely used in the last decades for gas-sensitive purposes [23,24,28,29,30]. They are ideal candidates for the optical detection of a number of gaseous and volatile analytes owing to their characteristic absorption, emission, charge transfer, and complexing properties as a result of their particular ring structure of conjugated double bonds [31]. They show a strong absorption in the UV–vis region, which is changed by the coordination of the analyte, thereby enabling their detection. At the same time, some porphyrins feature properties that make them effective for fluorescence gas sensors [30,32,33].

In early works, the host–guest approach was used to combine an inorganic matrix as host material with an organic sensing molecule as guest material [34]. Microstructured thin films made of TiO_2_ by electron beam physical vapour deposition in the configuration of glancing angle (GLAD-PVD) have shown good optical properties—such as optical transparency, high porosity, low refractive index, and controlled thickness—making them a great choice to be used as hosting matrices in optical sensors. The use of these microstructured materials aiming to enhance the material’s accessible surface area is a common approach that improves their functionality and can lead to a high number of applications. It has been reported that cationic porphyrins can be anchored to the negatively charged surface of TiO_2_ by electrostatic interaction forces [34]. Also, films prepared by the GLAD-PVD technique have been found to enhance the gas-sensing properties of the anchored porphyrins due to their open pores, which facilitate the access of the incoming gaseous molecules [24,34,35].

In this work, we present a gas sensor for the detection of gaseous ammonia and amines (butylamine, ethylenediamine, cadaverine, putrescine, and histamine). Our selection of analytes includes both solid and liquid amines to better explore the capabilities of the sensor. For this, we use composite films made of microstructured TiO_2_ and two different cationic porphyrins. The sensing mechanism consists of deprotonation (by the basic analytes) of the previously protonated porphyrin molecules. The analyte monitorisation is based on the changes that protonation (or deprotonation) produces in the absorption and emission spectra of the porphyrins, enabling the use of UV–vis and fluorescence spectroscopies.

## 2. Materials and Methods 

### 2.1. TiO_2_ Thin Film Preparation

Porous microstructures of TiO_2_ were prepared by physical vapour deposition in glancing angle configuration (GLAD-PVD) using an electron evaporation system [34,36,37]. Cut-to-size pieces (1.5 × 1.25 cm^2^) of glass (silicon wafer for specular reflectance Fourier-transform infrared (FT-IR)) were used as substrates, and kept at room temperature after preparation. The substrates were placed with a tilt angle of 70° with respect to the evaporation source. As a result, the angle between the tilted columns and the substrate surface was approximately 60°, which is in reasonably good agreement with the simplest empirical relation for GLAD, the tangent rule [38].

The obtained thin films were optically transparent, with a low reflectivity, and featured an open porous and tilted columnar microstructure with a thickness of 350 nm, exhibiting an elevated porosity (total pore volume of 49%) with void apertures on the surface in the form of mesopores (pore diameter > 2 nm), which also determined a relatively low refractive index value (1.79) [34]. Further details regarding film preparation and structural information can be found elsewhere [36].

### 2.2. Porphyrins and Reagents

**Figure 1:**
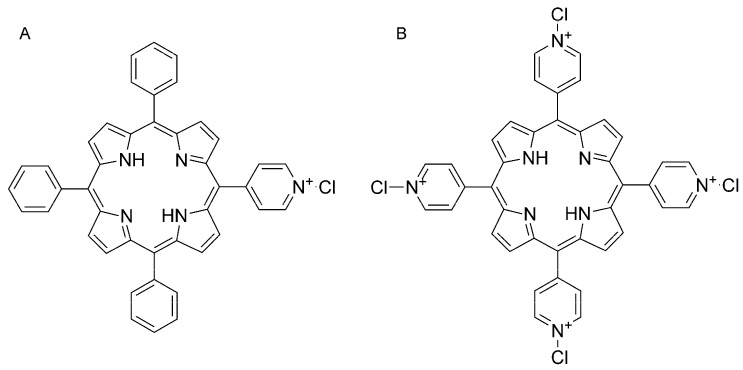
Schematic representation of the structure of the porphyrins (**A**) 5-(*N*-methyl 4-pyridyl)-10,15,20-triphenyl porphine chloride (MMPyP) and (**B**) meso-tetra (*N*-methyl 4-pyridyl) porphine tetrachloride (TMPyP).

The two porphyrins, 5-(*N*-methyl 4-pyridyl)-10,15,20-triphenyl porphine chloride (MMPyP, Figure 1A) and meso-tetra (*N*-methyl 4-pyridyl) porphine tetrachloride (TMPyP, Figure 1B), were supplied by Frontier Scientific (Logan, UT, USA) and used without further purification. Absolute ethanol from Sigma Aldrich was used as solvent. Hydrochloric acid, ammonia, butylamine, ethylenediamine, cadaverine, putrescine, and histamine were also purchased from Sigma Aldrich.

### 2.3. Composite Thin Film Preparation and Characterisation

Films were prepared by simple immersion of the TiO_2_ thin films into a 1 × 10^−5^ M ethanol solution of the corresponding porphyrin for 1 h. After this, the samples were washed with ethanol to remove any physisorbed dye molecules which were not anchored to the host matrix. Samples were then dried by blowing dry nitrogen onto the film surface for 5 min. The resulting composite thin films exhibited the characteristic yellowish colour of porphyrin thin films, suggesting that the dye molecules are in fact incorporated into the porous nanocolumnar structure and did not suffer any type of optical or structural change.

UV–vis spectra were recorded on a Cary100 Conc UV–visible spectrophotometer, except for the measurements in the gas chamber, which were performed with a UV–vis–NIR QE65000 detector (Ocean Optics Inc., Dunedin, FL, USA) and a DH2000 light source (Ocean Optics Inc., Dunedin, FL, USA). Fluorescence spectra were recorded using a Horiba Jobin-Yvon FluoroLog 3 spectrofluorometer (HORIBA Jobin-Yvon IBH Ltd., Glasgow, UK) operating in the front face mode. The emission spectra were excited with radiation of 420 nm for MMPyP and 430 nm for TMPyP using 2 and 4 nm slits for the excitation and emission monochromators, respectively. In the case of the measurements made in the gas chamber, we used 4 and 8 nm slits for the excitation and emission monochromators, respectively.

Time-resolved fluorescence decays were collected by time-correlated single-photon counting (TCSPC) using a FluoroLog 3. A NanoLED460 illuminating at 450 nm with a repetition rate of 1 MHz and a full width at half-maximum of 9 ns was used to excite the sample. The signals were recorded by using an IBH DataStation Hub photon counting module, and data analysis was performed by using DAS6 software (HORIBA Jobin-Yvon IBH Ltd., Glasgow, UK). The quality of fit was assessed by minimizing the reduced chi-squared function (χ_R2_) and by visual inspection of the weighted residuals.

Specular reflectance FT-IR spectroscopy was measured using a Jasco FT/IR-6200 spectrometer (Jasco Inc., Easton, PA, USA).

### 2.4. Gas Sensing System

The gas-detection system consisted of a cylindrical stainless steel homemade chamber. The system was designed to have the lowest possible volume in order to fill or empty the chamber quickly through a gas inlet and an outlet, having an internal cylinder-shaped volume of 12 cm diameter and 2 cm height (gas volume = 226 cm^3^).

The gas inlet was regulated by digital mass flow controllers (Bronkhorst High-Tech BV, Ruurlo, The Netherlands). A gas flow of 50 sccm was always used. The chamber contained two rectangular fused silica windows to externally connect two optical fibres to measure UV–vis absorption spectroscopy. One of them (50 μm diameter) was connected to the light source to illuminate the sample, while the other (600 μm diameter) delivered the transmitted light to the detector. Both optical fibres were connected to optical lenses to collimate the light. To measure fluorescence spectroscopy, the chamber had an additional square fused silica window with a bifurcated optical fibre, which transported the excitation light from the fluorimeter to the sample and, at the same time, delivered the emission light to the detector. By doing this, the system was designed to measure UV–vis and fluorescence simultaneously in the same gaseous environment with two samples, one for each type of spectroscopy.

We obtained the gaseous volatiles by bubbling dry nitrogen through a bottle containing the desired amines or a diluted solution of ammonia at room temperature. The solid amines (cadaverine, putrescine, and histamine) were previously melted inside a flask and the saturated vapours were carried to the chamber. The volatile concentrations were calculated through their vapour pressure at the corresponding temperature and obtained by using the mass flow controllers to precisely dilute the volatiles with dry nitrogen.

Prior to the exposure to amines or ammonia, the samples were protonated by their exposure to an acidic atmosphere. For this purpose, HCl gas was directed into the chamber until complete protonation of the samples. After this, the samples were ready for the gas-detection experiments.

## 3. Results and Discussion

### 3.1. Optical Properties of MMPyP and TMPyP before and after Protonation

**Figure 2:**
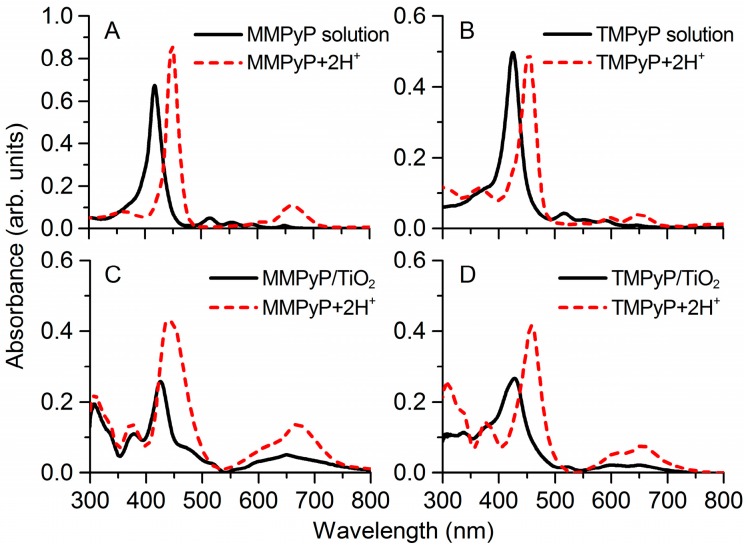
Absorbance spectra of (**A**,**C**) MMPyP and (**B**,**D**) TMPyP in ethanol solution and in TiO_2_ solid films before (solid black line) and after protonation (dashed red line).

The UV–vis spectra of MMPyP and TMPyP solutions showed the typical profile of the monomeric state of these porphyrins, with their Soret bands centred at 417 nm and 425 nm, respectively (Figure 2A,B). When anchored to the TiO_2_ films, both porphyrins featured a shifting and broadening of their Soret band with respect to solution (Figure 2C,D). In the case of MMPyP, there was a clear 9 nm red-shift of the Soret band, while in the case of TMPyP, a shoulder appeared at 415 nm, meaning a partial 10 nm blue-shift with respect to its monomeric state. These results indicate different aggregation states of the porphyrins into the TiO_2_ matrix.

The shifting and broadening of the absorption spectra of aggregated species in thin films, relative to the monomeric spectrum in solution, has been interpreted by application of exciton models, such as the point dipole model proposed by McRae and Kasha [39] or the extended dipole model proposed by Kuhn and collaborators [40,41]. Excellent discussions and comparisons between them can be found in the literature [42,43,44]. In summary, two types of molecular aggregates can be formed according to the relative displacement of their interacting dipoles. A J-aggregate is formed when Coulomb attraction forces between the interacting dipoles outweigh repulsion (giving rise to a lower energy band gap), which is the case for stacked porphyrins, where the central rings are slipped with respect to each other. Conversely, H-aggregates result from the face-to-face arrangement of porphyrin macrocycles, where repulsion is predominant [45].

The 9 nm red-shift of the Soret band of the MMPyP/TiO_2_ spectrum can be attributed to J-type aggregation of the MMPyP porphyrins in the TiO_2_ matrix [46]. On the other hand, the 10 nm blue-shifted shoulder observed in the TMPyP/TiO_2_ spectrum can be attributed to a partial formation of H-aggregates. It has been reported that TMPyP molecules anchored to an anionic matrix may adopt a dimer configuration as H-aggregates, in which the parallel rings of the molecules are twisted by 45° with respect to each other [46] (see Figure S1 in the Supplementary Materials), resulting in a 10 nm blue-shift in the Soret band of TMPyP after dimer formation (as predicted by the extended dipole model) [40,41]. The different aggregation states proposed here for the two porphyrins can be explained in terms of their different number of interacting points (positive charges) with the TiO_2_ matrix. In this way, while MMPyP molecules have only one methyl pyridyl anchoring group and, consequently, can be found nearly perpendicular with respect to the oxide surface, TMPyP configuration allows for an anchoring by its four positive groups, resulting in the molecule lying parallel to the matrix surface. These two configurations can lead to different forms of aggregation, where the perpendicular MMPyP molecules can aggregate with a certain displacement of their respective rings giving rise to J-type aggregates, while the parallel orientation of the TMPyP molecules may lead to H-aggregates in the form of dimers similar to those described above [47] with their respective rings twisted by 45º, thus allowing the interaction of their eight positive charges with the TiO_2_ matrix. This different aggregation based on the different number of anchoring points has also been described for mono- and tetra-substituted carboxyphenyl porphyrins in the same TiO_2_ matrix [48]. This highlights the importance of the type of substituent in infiltrated dyes and its influence on the molecular aggregation, which can be critical to its potential functionality into the composite.

After their exposure to HCl gas, both MMPyP and TMPyP suffered an intense red-shift in their Soret bands, and the number of Q-bands turned from four into two as a result of the protonation of the molecules (Figure 2). This phenomenon was observed both in solution and in the solid films. The structural change that occurs in a porphyrin molecule when it is protonated is well known and basically consists of a flattening deformation. The molecule adopts a saddle-type structure in which the two pyrrole rings with deprotonated nitrogen atoms point upwards, while the two protonated ones point downwards [49]. As a consequence, the Soret band undergoes a wavelength red-shift of about 30 nm and, due to a higher symmetrical configuration of the protonated ring, the original four Q-bands with D_2h_ molecular symmetry turn into two Q-bands with *D_4h_* molecular symmetry [35].

**Figure 3:**
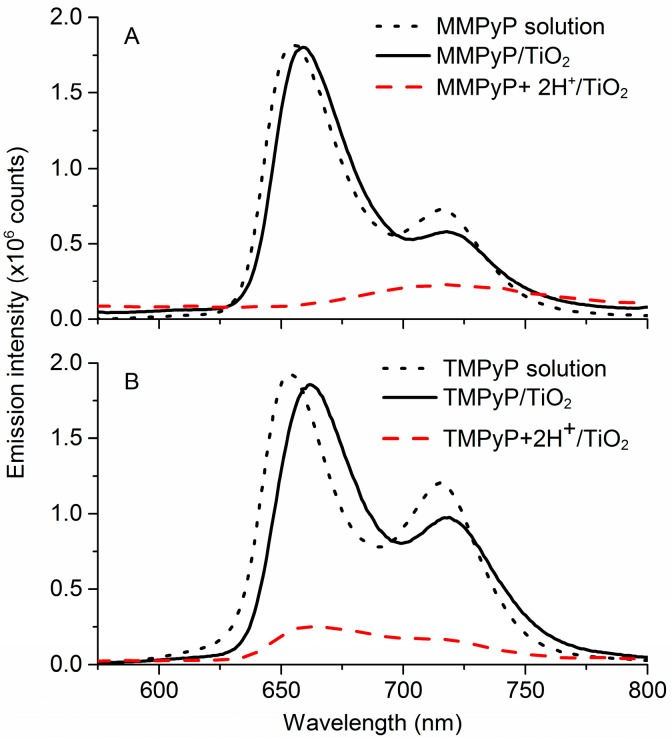
Emission spectra for (**A**) MMPyP/TiO_2_ and (**B**) TMPyP/TiO_2_ films before (solid black line), and after protonation (dashed red line). The corresponding spectra in ethanol solution are also shown (dotted black line).

MMPyP/TiO_2_ and TMPyP/TiO_2_ fluorescence emission spectra were also measured before and after protonation (Figure 3). The spectra of both porphyrins presented two well-differentiated bands corresponding to the degeneracy of the lowest singlet configuration of the porphyrins [50]. The shape of the fluorescence spectra of the porphyrins in the TiO_2_ matrix was similar to those in ethanol solution, indicating that the polarity of the solution was similar to that of the composite.

Once protonated, the emission of the porphyrin/TiO_2_ composite films suffered a drastic decrease, which is in good agreement with previous research [34,35]. In particular, Kalimuthu and John reported that the high decrease of the emission intensity is due to the loss of flattening of the porphyrins [51]. The corresponding peaks for the two porphyrins before and after protonation, along with the isosbestic point, are indicated in Table 1. The emission changes under protonation were also confirmed by TCSPC, which showed an effective luminescence deactivation associated to the protonation process for the two porphyrins (Figure S2 in the Supplementary Materials).

### 3.2. Response to Ammonia

The gas-sensing properties of the porphyrin/TiO_2_ composites were first investigated by exposing the previously protonated films to ammonia vapours. During exposure to the analyte, the emission changes were recorded and expressed in the form of kinetic responses at the wavelength of maximum change (λ = 660 nm). Emission was chosen over absorbance as sensing parameter because of the higher relative change that can be obtained with this technique, resulting in a more sensitive sensor.

**Figure 4:**
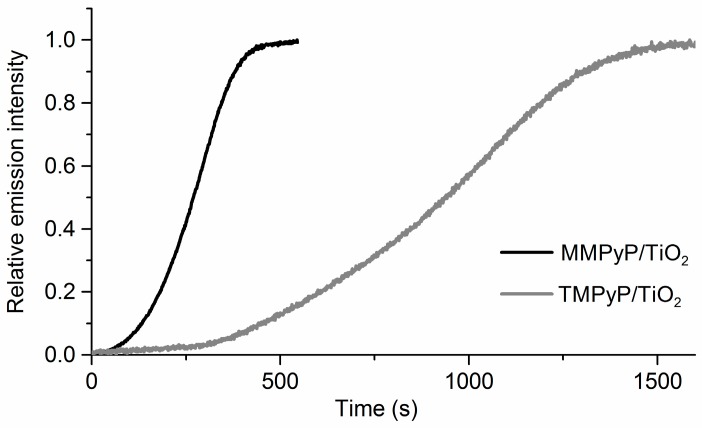
Normalised kinetic response at 660 nm of the protonated MMPyP/TiO_2_ (black line) and TMPyP/TiO_2_ (grey line) composites to 600 ppm of ammonia vapours.

The fluorescence emission of both porphyrins experienced an increase as a consequence of the recovery of the unprotonated state of the porphyrin in the presence of ammonia vapours (Figure 4). It is worth noting that simply flushing N_2_ does not lead to the recovery of the unprotonated state of the porphyrins, indicating the stability of their protonated form. No influence of the humidity was observed. The MMPyP/TiO_2_ composite showed a much faster response than that of TMPyP/TiO_2_. This can be quantified by calculating their respective response times, defined as the time required for the sensor output to change from its initial state to a final settled value. There are several ways of stating response times, such as *t_c_*, *t_50_*, or *t_10–90_*, which are the times taken for the sensor to reach 63%, 50%, and from 10% to 90% of its final value, respectively [52]. The calculated response times for the exposure to 600 ppm ammonia indicated that the MMPyP/TiO_2_ sensor (*t_c_* = 301 s; *t_50_* = 270 s; *t_10–90_* = 247 s) was always more than three times faster than the TMPyP/TiO_2_ composite (*t_c_* = 1044 s; *t_50_* = 938 s; *t_10–90_* = 836 s). This significant difference may be attributed to the different arrangement of the two porphyrins in the TiO_2_ matrix, as proposed above, after the analysis of the UV–vis spectra. In this way, the partial face-to-face stacking of the TMPyP derivative, with its four positive groups interacting with the TiO_2_ surface, could hinder the access of the analyte molecules to the porphyrin ring, while the more open geometry of the MMPyP molecules, anchored by its only phenyl pyridyl group, would facilitate the analyte interaction.

**Figure 5:**
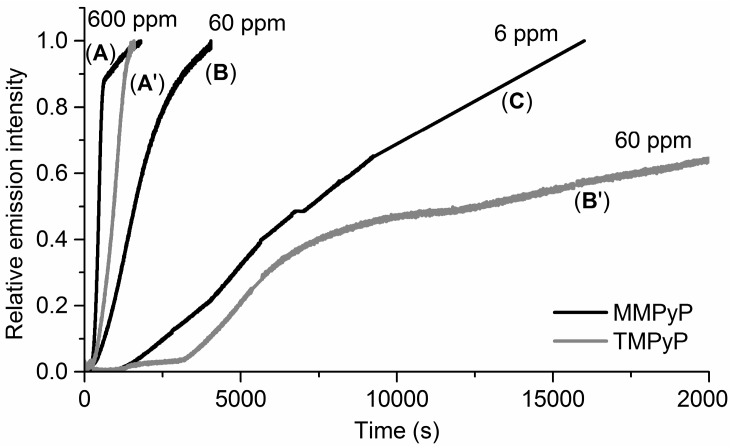
Kinetic responses of the MMPyP/TiO_2_ (**A**–**C**) and TMPyP/TiO_2_ (**A’**,**B’**) composites to different concentrations of ammonia vapours. (**A**,**A’**) correspond to 600 ppm; (**B**,**B’**) correspond to 60 ppm and (**C**) corresponds to 6 ppm.

Figure 5 shows the sensor response to different concentrations of ammonia vapours. In all cases, the response time increases as the analyte concentration decreases. However, significant differences can be observed in the behaviour of both derivatives. While the MMPyP/TiO_2_ composite gives reasonable response times to 600, 60, and 6 ppm of NH_3_, the TMPyP/TiO_2_ system shows a considerably slower response to 60 ppm and no response to 6 ppm. The explanation of this result can be found again in the different attachments of the two porphyrins to the TiO_2_ matrix. In this way, the perpendicular arrangement of the MMPyP molecules with respect to the TiO_2_ would keep their rings more separated from the matrix and hence more accessible to the volatiles. On the other hand, the TMPyP molecules would be placed parallel to the matrix with their central ring close to the oxide surface, hindering analyte access. It is worth noting that the protonation (HCl) and subsequent deprotonation (NH_3_) of both porphyrins in solution resulted in immediate changes, indicating that the different response times, once in the film, are determined by their different aggregation geometries and not because of differences in the sensing capabilities of the molecules.

The limit of detection (LOD) was calculated for the exposure of both composites to ammonia vapours according to the corresponding signal to noise ratio (LOD = 3 × signal-to-noise ratio—SNR). MMPyP/TiO_2_ exhibited a higher slope than TMPyP/TiO_2_, suggesting an enhancement of the sensor sensitivity. The relative standard deviation (RSD) was also calculated using the protonated composites as the initial point and exposing them to ammonia vapours. In both cases, very low values were obtained with 2.3% for MMPyP/TiO_2_ and 3.7% for TMPyP/TiO_2_. These and other relevant analytical parameters are summarised in Table 2.

### 3.3. Selectivity and Repeatability of the Sensing System

**Figure 6:**
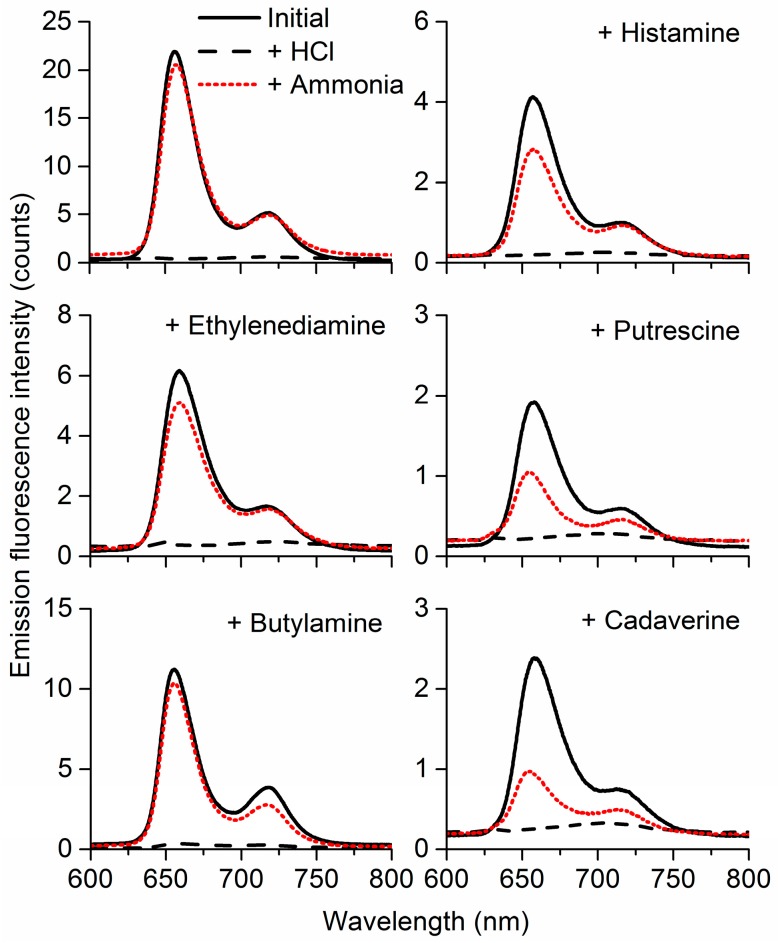
MMPyP/TiO_2_ spectra before any exposure (solid black line), after protonation with HCl gas (dashed black line), and after exposure of its protonated form to ammonia and amine vapours (dotted red line).

Given the overall better sensing performance of the MMPyP composites, we further analysed its gas sensing properties towards ammonia and amines. The selectivity of MMPyP/TiO_2_ was studied by exposing the previously protonated films to vapours of ammonia, histamine, ethylenediamine, putrescine, butylamine, and cadaverine. As a result of the exposure, all composites showed a substantial recovery of the original emission spectrum caused by the deprotonation of the porphyrins (Figure 6). This recovery was found to be considerably higher for liquid amines and ammonia than for solid amines, which can be related to their different volatilities. Partial condensation of the solid amines on the walls of the chamber might also have contributed to the lower responses towards these amines. Nonetheless, to ensure that the vapours generated from solid amines were reaching the sample, we exposed the composite films to the headspace of a sealed flask with the corresponding melted amine and then recorded their emission spectra, resulting in similar changes as those obtained from the gas-sensing system and indicating that a process of massive condensation was not the main cause for the lower response. In any case, the degree of recovery was different for each of the analytes, suggesting a selective behaviour of the protonated MMPyP/TiO_2_ composite films towards these compounds.

**Figure 7:**
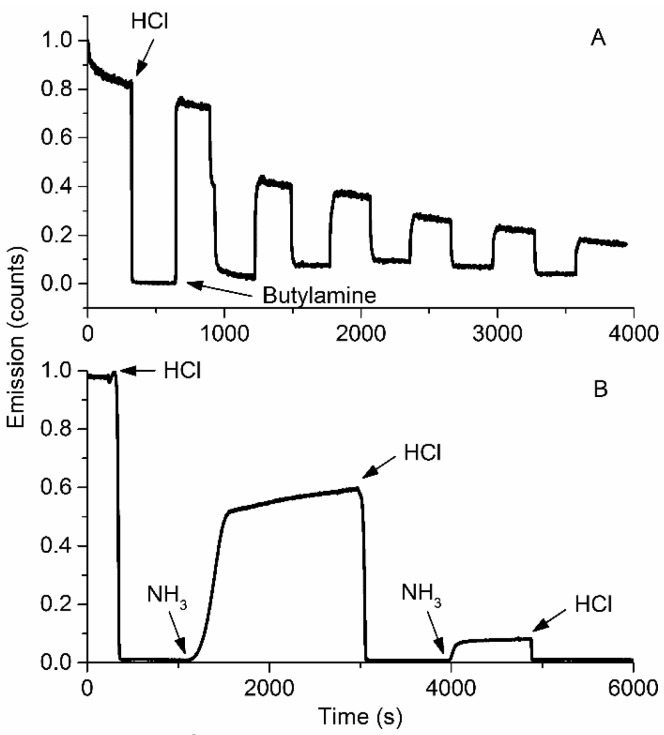
Kinetic response of the MMPyP/TiO_2_ composite to saturated vapours of (**A**) butylamine and (**B**) ammonia. Arrows indicate the beginning of the exposure to the indicated gas.

To account for the repeatability of the system, we exposed MMPyP/TiO_2_ composites to several cycles of protonation with hydrogen chloride gas and subsequent deprotonation with saturated vapours of butylamine or ammonia (Figure 7A). The intensity of the signal decreased progressively with the successive exposure/recovery cycles. The use of ammonia instead of butylamine led to the same result with an even more dramatic decrease of intensity (Figure 7B; full spectra evolution can be found in Figure S3 in the Supplementary Materials). A similar behaviour was observed for the TMPyP/TiO_2_ composite.

**Figure 8:**
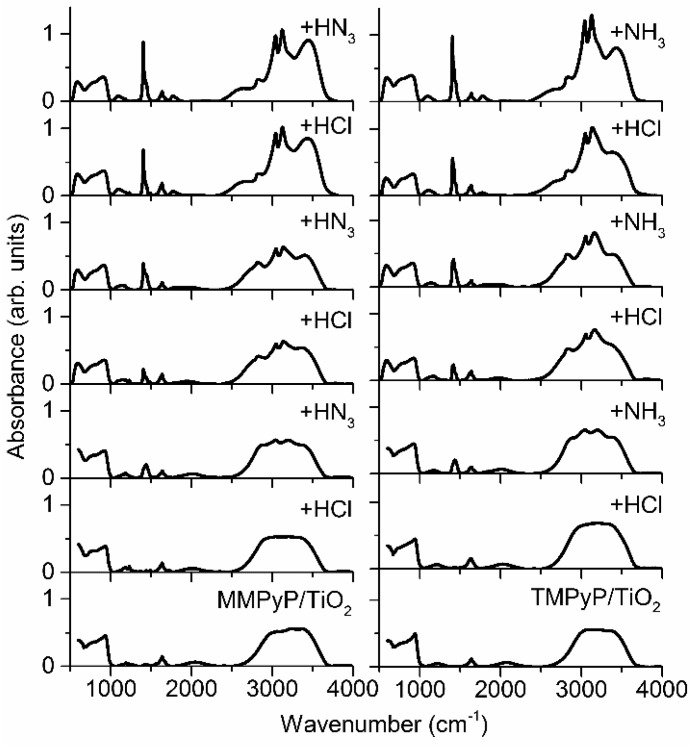
Infrared spectra of the MMPyP/TiO_2_ (**left**) and TMPyP/TiO_2_ (**right**) composites under successive exposure cycles to HCl and NH_3_. Number of exposures increases from bottom (unexposed samples) to top.

We hypothesised that this decrease of the fluorescence emission intensity could be due to the formation of ammonium salts inside the films after the successive cycles, which also caused an increase of the scattering after each cycle (see Figure S3 in the Supplementary Materials). In order to get a better understanding of this loss of emission intensity in the successive cycles of response/recovery, specular reflectance FT-IR spectra were performed on both composites after alternate exposures to HCl and NH_3_ vapours (Figure 8). MMPyP/TiO_2_ and TMPyP/TiO_2_ spectra were characterised by the existence of typical bands corresponding to the symmetric and asymmetric stretching modes of the pyrrole ring (ν(C–H), ν(C=C), and ν(C=N)) within the meso-tetraphenylporphyrin macrocycle in the 700–1500 cm^−1^ range [53]. Also, a peak at 1637 cm^−1^ indicated the presence of free TiO_2_ molecules in the columnar film, and a broad region around 2800–3500 cm^−1^ indicated where ν(C–H) bonds were located [53].

Both composites showed similar behaviour after the successive cycles of exposure to HCl and NH_3_. Three new bands appeared at 1403, 3041, and 3127 cm^−1^ that invariably grew with the successive exposures. As hypothesised, these bands corresponded to the formation of ammonium chloride (see Figure S4 in the Supplementary Materials). A broad band was also observed at 3442 cm^−1^, corresponding to ν(O–H) stretch and H-bonds due to the aqueous environment [54]. The formation of the ammonium chloride salts led to the degradation of the optical quality of the films, progressively reducing their transparency. This fact provoked the loss of emission intensity observed in the repeatability study, suggesting that the sensor itself probably suffered no degradation after several cycles of protonation–deprotonation.

## 4. Conclusions

Two *N*-methyl 4-pyridyl cationic porphyrins were anchored to nanostructured columnar thin films of TiO_2_ by electrostatic interaction, and their sensing capability towards ammonia and amines has been investigated by following the emission changes of the protonated porphyrins in the presence of the gaseous analytes.

Protonation of the porphyrins was carried out by exposing the films to saturated HCl vapours with the typical changes in intensity and wavelength of the corresponding absorbance and emission spectra. The absorbance spectra of the composite films also showed some shifting and broadening of the Soret band with respect to solution, which is indicative of intermolecular aggregation. These changes were, however, different for the two porphyrins, with the monocationic derivative showing J-aggregation while the tetracationic exhibited some degree of H-aggregation. These differences are consistent with the molecular structure of two porphyrins. In this way, MMPyP is anchored to the TiO_2_ matrix with its only cationic peripheral group giving rise to a perpendicular arrangement with respect to the oxide surface. On the other hand, TMPyP interacts with the matrix, with its four positive charges more likely to be in a parallel orientation with respect to the TiO_2_ columnar surface, with a second porphyrin molecule being anchored to the oxide on top of the first one. These different arrangements produce slipped aggregates (J-aggregation) in the case of MMPyP and face-to-face interaction (H-aggregates) in the case of TMPyP.

The two composite films were exposed to different concentrations of ammonia and butylamine vapours after previous protonation of the porphyrins. Both films showed significant emission changes in the presence of concentrated analytes, however, the monocationic derivative exhibited a much faster response and lower detection limit. This behaviour has been associated to the different arrangements of the two porphyrins in the microcolumnar film. In this way, the more accessible ring of the monocationic derivative with its only anchoring point allows easier and faster interaction with analytes.

The overall better response of MMPyP/TiO_2_ films suggests they are a better choice for the detection of ammonia and amines than TMPyP/TiO_2_ films. The exposure of the monocationic derivatives to ammonia and five different amines after previous protonation revealed their potential to selectively detect these compounds

Finally, the repeatability of the sensor was investigated by performing consecutive exposure–recovery cycles with a significant loss of fluorescence emission intensity. Specular reflectance FT-IR revealed the formation of ammonium salts inside the films after complete cycles of exposure to HCl and analyte, which has been found to be responsible for the progressive loss of intensity in the successive cycles, mainly due to degradation of the optical quality of the films that gradually lost transparency.

## Figures and Tables

**Table 1:** Position of the main peaks and isosbestic points in the absorption and emission intensity spectra for MMPyP and TMPyP in solution and in TiO_2_ films before and after protonation.

	Absorbance Peaks					Fluorescence Peaks		
	B(0,0)	Q_y_(1,0)	Q_y_(0,0)	Q_x_(1,0)	Q_y_(0,0)	Isosbestic Point	Q(0,0)	Q(0,1)
MMPyP solution	415	515	554	591	648	434	653	715
MMPyP+2H^+^ solution	448		556	608	659	434		
MMPyP/TiO_2_	426	518	558	596	649	431	657	718
MMPyP+2H^+^/TiO_2_	440			611	670	431	718	
TMPyP solution	425	516	551	590	647	438	653	714
TMPyP+2H^+^ solution	453		551	600	652	438		
TMPyP/TiO_2_	427	523	560	600	651	443	662	717
TMPyP+2H^+^/TiO_2_	458			612	654	443	662	717

**Table 2:** Analytical characteristics obtained from the calibration curves of MMPyP/TiO_2_ and TMPyP/TiO_2_ under ammonia exposure.

	MMPyP/TiO_2_	TMPyP/TiO_2_
Intercept (counts × 10^4^)	1.7 ± 0.2	9.7 ± 1.2
Slope (counts/ppm × 10^2^)	10.4 ± 0.6	3.6 ± 0.4
Regression coefficient (r)	0.99	0.99
Standard deviation of residuals (S_y/x_)	14.1	17.6
Limit of detection (ppm)	0.05	0.16
Relative standard deviation (%)	2.3	3.7
Measurement wavelength (nm)	660	660

## Supplementary Materials

The following are available online at http://www.mdpi.com/1424-8220/17/1/24/s1, Figure S1: Schematic representation of aggregate formation for: (A) MMPyP (J-aggregate) and (B) TMPyP (H-aggregate) porphyrins; Figure S2: Fluorescence decay curves in natural and protonated state of: (A) MMPyP solution in ethanol, (B) TMPyP solution in ethanol, (C) MMPyP/TiO_2_ composite and (D) MMPyP/TiO_2_ composite; Figure S3: Fluorescence emission spectra of MMPyP/TiO_2_ composite after successive exposure cycles to HCl and ammonia. The base line moving upwards after each cycle was attributed to increased scattering by the formation of ammonium salts; Figure S4: Infrared spectra of MMPyP/TiO_2_ and TMPyP/TiO_2_ after three cycles of protonation with HCl and exposure to ammonia. The chloride ammonium infrared spectrum is included for comparison.
