# Supplementary material for: Optical Gas Sensing of Ammonia and Amines Based on Protonated Porphyrin/TiO2 Composite Thin Films
Source: Sensors (Basel). 2016 Dec 23;17(1):24. doi: 10.3390/s17010024 (PMC5298597; doi:10.3390/s17010024)
Supplement: Supplementary file 1 [file sensors-17-00024-s001.pdf]

Supplementary Materials: Optical Gas Sensing of Ammonia and Amines Based on Protonated Porphyrin/TiO_2 Composite Thin Films

Pedro Castillero, Javier Roales, Tânia Lopes-Costa, Juan R. Sánchez-Valencia, Angel Barranco, Agustín R. González-Elipe and José M. Pedrosa

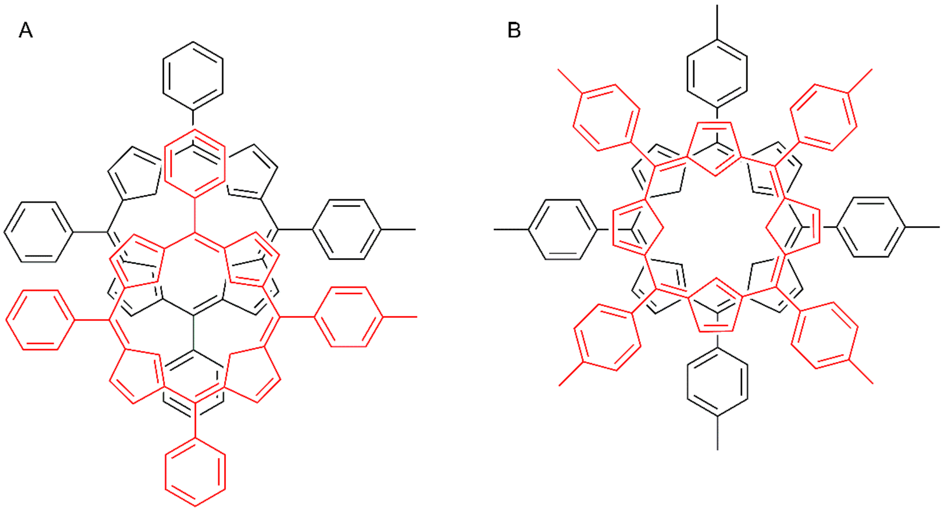

Figure S1: Schematic representation of aggregate formation for: (**A**) MMPyP (J-aggregate) and (**B**) TMPyP (H-aggregate) porphyrins.

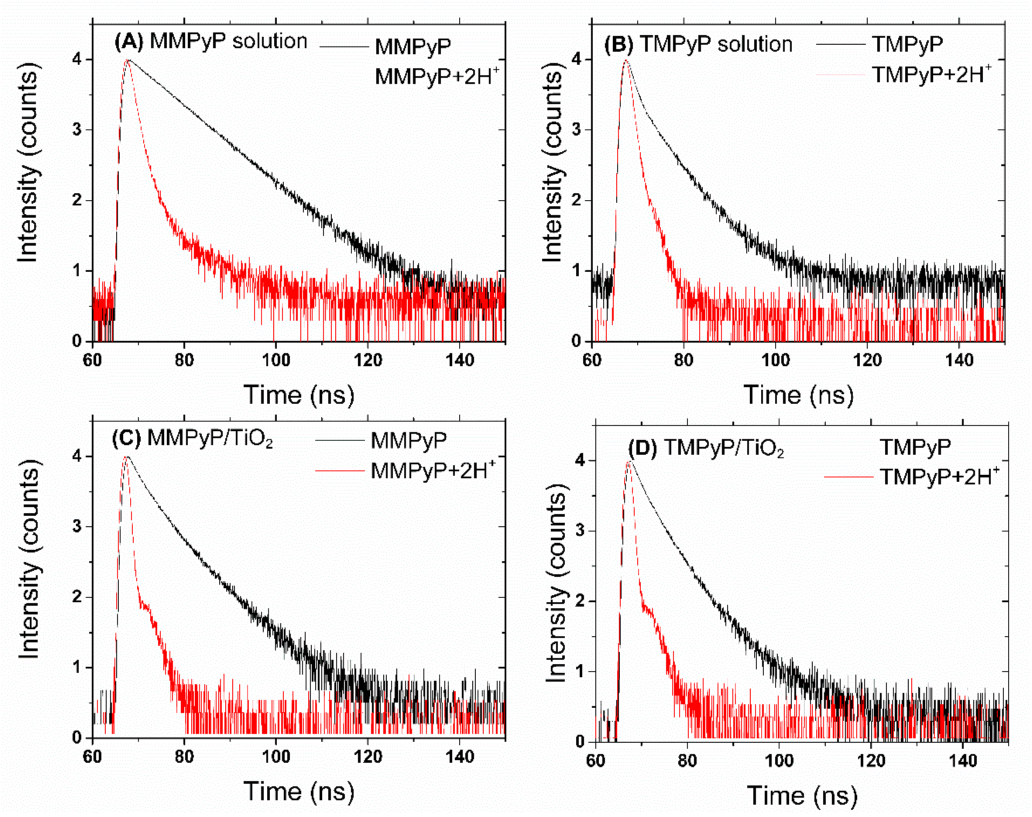

Figure S2. Fluorescence decay curves in natural state (black line) and in protonate state (red line) of: (**A**) MMPyP solution in ethanol; (**B**) TMPyP solution in ethanol; (**C**) MMPyP/TiO_2 composite and (**D**) MMPyP/TiO_2 composite.

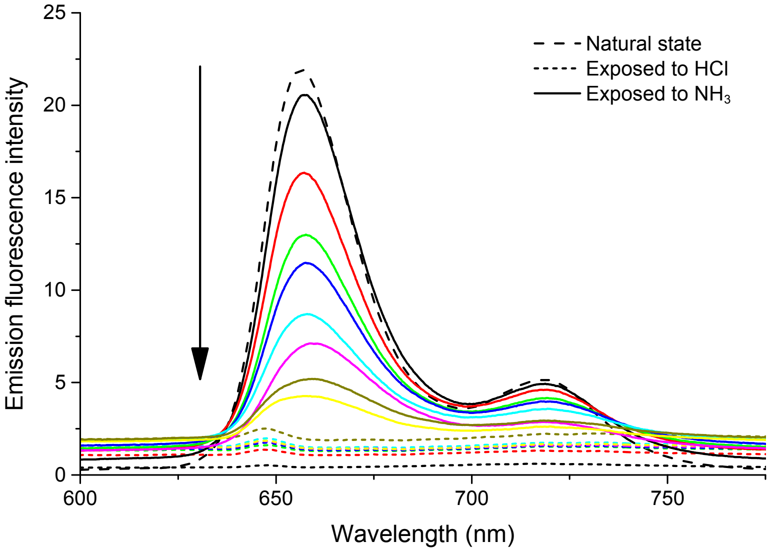

Figure S3. Fluorescence emission spectra of MMPyP/TiO_2 composite after successive exposure cycles to HCl and ammonia. The base line moving upwards after each cycle was attributed to increased scattering by the formation of ammonium salts.

Bands of Chloride ammonium can be identified in the FT-IR spectra of the samples exposed to cycles of HCl/NH_3 composites as shown in the Figure S4. The peak at 1636 cm^{-1} corresponds to the free TiO_2 molecules of the columnar film [1] and the peak in 3500 cm^{-1} corresponds to water environment [2].

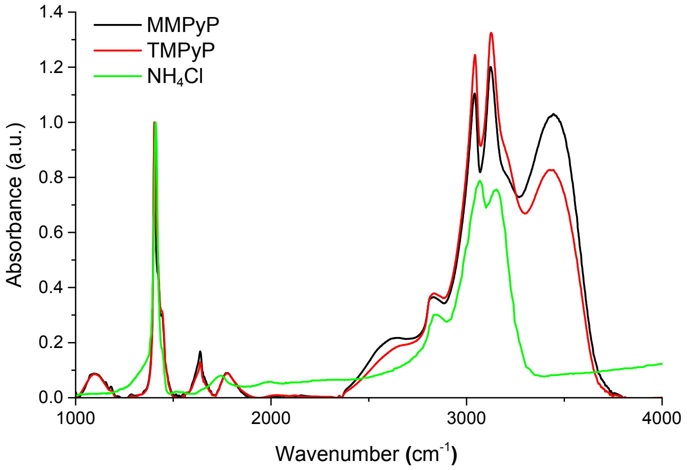

Figure S4. Infrared spectra of MMPyP/TiO_2 (black line) and TMPyP/TiO_2 (red line) after three cycles of protonation with HCl and exposure to ammonia. The chloride ammonium infrared spectrum (green line) is included for comparison.

References

1. Roales, J.; Pedrosa, J. M.; Cano, M.; Guillén, M. G.; Lopes-Costa, T.; Castillero, P.; Barranco, A.; Gonzalez-Elipe, A. R. Anchoring effect on (tetra)carboxyphenyl porphyrin/TiO_2 composite films for VOC optical detection. *RSC Adv.* **2014**, *4*, 1974–1981.
2. Max, J.-J.; Chapados, C. Aqueous ammonia and ammonium chloride hydrates: Principal infrared spectra. *J. Mol. Struct.* **2013**, *1046*, 124–135.
